# Applications of IMAT in cervical esophageal cancer radiotherapy: a comparison with fixed‐field IMRT in dosimetry and implementation

**DOI:** 10.1120/jacmp.v12i2.3343

**Published:** 2011-01-13

**Authors:** Yong Yin, Jinhu Chen, Ligang Xing, Xiaoling Dong, Tonghai Liu, Jie Lu, Jinming Yu

**Affiliations:** ^1^ School of Information Science and Engineering Shandong University Jinan China; ^2^ Department of Radiation Physics Shandong Cancer Hospital and Institute Jinan China; ^3^ Department of Radiation Oncology Shandong Cancer Hospital and Institute Jinan China

**Keywords:** intensity‐modulated radiotherapy, intensity‐modulated arc radiotherapy, dosimetry, esophageal neoplasms

## Abstract

This study aimed to compare fixed‐field, intensity‐modulated radiotherapy (f‐IMRT) with intensity‐modulated arc therapy (IMAT) treatment plans in dosimetry and practical application for cervical esophageal carcinoma. For ten cervical esophageal carcinoma cases, f‐IMRT plan (seven fixed‐fields) and two IMAT plans, namely RA (coplanar 360° arcs) and RAx (coplanar 360° arcs without sectors from 80° to 110°, and 250° to 280°), were generated. DVHs were adopted for the statistics of above parameters, as well as conformal index (CI), homogeneity index (HI), dose‐volumetric parameters of normal tissues, total accelerator output MUs and total treatment time. There were differences between RAx and f‐IMRT, as well as RA in PTV parameters such as HI, $V_{95\%}$ and $V_{110\%}$, but not in CI. RAx reduced lung $V_{5}$ from (50.9%± 9.8% in f‐IMRT and (51.4%± 10.8% in RA to (49.3%± 10.4% in RAx (p<0.05). However, lung $V_{30},V_{40},V_{50}$ and MLD increased in RAx. There was no difference in the mean heart dose in three plans. Total MU was reduced from 1174.8±144.6 in f‐IMRT to 803.8±122.2 in RA and 736.2±186.9 in RAx (p<0.05). Compared with f‐IMRT, IMAT reduced low dose volumes of lung and total MU on the basis of meeting clinical requirements.

PACS numbers: 87.55.D, 87.55.dk, 87.55.ne

## I. INTRODUCTION

Definitive chemoradiotherapy is the standard of care for cervical esophageal cancer.^(^ ^1^ ^–^ ^3^ ^)^ However, because of complexity of anatomic structures in head and neck, conventional radiotherapy often results in inadequate tumor dosage and/or acute and chronic normal tissue toxicity. In recent years, many studies demonstrated the dosimetric advantages of fixed‐field, intensity‐modulated radiotherapy (f‐IMRT) compared with conventional or 3D conformal therapy in sparing organs at risk (OAR) as well as improving tumor dose coverage.^(^ ^4^ ^–^ ^6^ ^)^ But the long treatment time of f‐IMRT would enhance the influences of uncertain factors during the treatment, such as body displacement, organ motion and discomfort of the patients. The clinical applications of intensity‐modulated arc radiotherapy (IMAT), a combination of intensity‐modulated radiotherapy and arc radiotherapy, has come to reality with the hardware and software developments, such as RapidArc with Varian Eclipse v8.6 treatment planning system (Varian Medical Systems, Palo Alto, CA). In RapidArc plans, a series of apertures are generated through serial movements of dynamic MLC while the accelerator gantry rotates continuously, and accompanied by variable dose rate. The dynamic parameters, including variations of dose rate, gantry rotation speed, leaf motion speed and gantry position, can be synthesized jointly to create IMRT dose distributions. For dose calculation, the Anisotropic Analytical Algorithm (AAA) was used. It showed that the calculation of the dose distribution can be performed with a clinically acceptable accuracy using a resolution of 2.5 mm or better.^(7)^ Court et al.^(8)^ concluded that pencil‐beam algorithm, used in chest tumor dose calculation, would underestimated the lung dose. Therefore, both f‐IMRT plan and RapidArc plans used AAA for dose calculation.

Previous dosimetric studies indicated that IMAT was superior or equivalent to f‐IMRT. Palma et al.^(9)^ reported that IMAT was better than f‐IMRT for the rectum and femoral head in prostate cancer cases. Cozzi et al.^(10)^ concluded that RapidArc plan in cervix uteri cancer had improvements in sparing organs at risk such as intestine and bladder with uncompromised target coverage, in comparison with f‐IMRT. At the same time, there's a common observation of reduction of total MU in these studies. In this study, f‐IMRT and RapidArc plans were compared for cervical esophageal cancer cases, and the application of the techniques was discussed.

## II. MATERIALS AND METHODS

### A. Patients

Ten patients with pathological proven cervical esophageal squamous cell cancer and who received radiation therapy in our hospital from November 2009 to February 2010 were enrolled in the study. There are three cases with $T_{4}N_{0}M_{0}$ and seven cases with $T_{4}N_{1}M_{0}$ according to the AJCC staging system (AJCC, 1997). CT simulation (Philips Brilliance 16) was conducted and the patient underwent immobilization using thermoplastic facemask at supine position. The scan was from C2 to L1 with 3 mm thickness.

### B. Radiation therapy plans

For each case, bilateral supraclavicular lymphatic drainage area, primary tumor and esophageal/paratracheal lymph nodes were segmented as gross tumor volume (GTV) and clinical tumor volume (CTV) by radiologists and radiation oncologists jointly. Then planning target volume (PTV) was generated from GTV and CTV plus margins (GTV: 3–5 cm superior and inferior, 1.5–2 cm lateral; and CTV: 0.5–1 cm in all directions), based on clinical experiences and previous studies,^(^ ^11^ ^–^ ^13^ ^)^ as well as the location of tumor. The volume of PTV on average was 297.9±104.2 cc. The Varian Eclipse v8.6 treatment planning system (Varian Medical Systems, Palo Alto, CA) and the Varian Trilogy accelerator were used. For each case, one f‐IMRT plan and two RapidArc plans (i.e., RA and RAx) were generated. Six MV X‐ray and AAA were used for dose calculation. Initiation total prescription dose of all the plans was 60 Gy (2 Gy/fraction, 30f). Plan 100% dose level was normalized at PTV mean dose. The planning optimization objectives were set as follows: 98% of the volume of PTV reached 95% of the prescription dose with 10% of the volume of PTV not exceeding 110% the prescription dose, maximum spinal cord dose no more than 45 Gy, total lung $V_{5}<45\%,V_{10}<35\%$, and $V_{20}<20\%$. It was required that there was no cold spot in PTV and no hot spot in esophagus wall. It should be noted that this study was only a dosimetric comparison. Therefore, despite the fact that the cases had received chemotherapy, all plans were generated following the objectives. During the optimization, the dose constraints were adjusted down as low as possible, due to the diversity of PTV or total lung volume – though some cases could not reach the objectives.

#### B.1 f‐IMRT plan

Step‐and‐shoot technique was adopted, with intensity level 20, dose rate 300 MU/min, and seven coplanar fields set at 0°, 51°, 103°, 154°, 206°, 257° and 309°.

#### B.2 RA

Two coplanar full arcs were set counter‐clockwise 179° to 181° with collimator angle 30°, and clockwise 181° to 179° with collimator angle 330°. The highest dose rate was 600 MU/min, and maximum gantry rotation velocity 4.8 deg/min. In the plan, a full rotation field (or arc) consists of 177 apertures (one aperture per 2 deg), each of which has a different dose rate in order to deliver different MU and rotates at maximum speed. In the case that, at some angle, one aperture could not meet the dose requirement with the maximum dose rate, the gantry rotation velocity will slow down.

#### B.3 RAx

Two avoidance sectors (from 80° to 110°, and 250° to 280°) were set based on RA plan. When gantry rotated in the avoidance sectors, accelerator beamed off with no X‐ray output.

### C. Plan evaluation

The cumulative DVHs were generated for evaluation and comparison. For PTV, the values of $D_{98\%}$ and $D_{2\%}$ (dose received by the 98% and 2% of the PTV volume) were defined as metrics for minimum and maximum doses. Parameters below were recorded and compared:

a) PTV: $D_{98\%},D_{2\%},V_{95\%},V_{110\%}$, HI (heterogeneity index) and CI (conformal index)
b) total lung: $V_{5}$ (the volume receiving 5 Gy), $V_{10},V_{20},V_{30},V_{40},V_{50}$ and mean lung dose (MLD)
c) B‐P: $V_{3},V_{5}$ and $V_{10}$. B‐P was defined as the patient's volume covered by the CT scan minus the PTV that represented healthy tissues.
d) mean received dose for heart
e) total MU and total treatment time, which was defined as the time from the beginning of beam on to the end of total MU delivery (In this study, all 30 plans were delivered actually without patients on board in order to count the real treatment time.)

The paired, two‐tails Student's t‐test was applied by using SPSS 13.0 software. $\text{HI}=(D_{2}-D_{98})/\;\text{prescription dose}*100\%;$ ^(14)^ and $\text{CI}=V_{t,\text{ref}}/V_{t}*V_{t,\text{ref}}/V_{\text{ref}}$. (Note: $V_{t}=$ target volume, $V_{t,\text{ref}}$ = target volume wrapped by reference isodose, $V_{\text{ref}}=$ all volume wrapped by reference isodose^(15)^). The value of CI is between 0 and 1, and the closer the value is to 1 it presents a better conformity.

## III. RESULTS

### A. PTV coverage

As summarized in Table 1, statistical difference of HI, $D_{2\%}$ and $V_{110\%}$ were observed as comparison of RAx with f‐IMRT and RA (p<0.05), which meant that the high‐dose volume of RAx enlarged. HI in RAx increased from 13.19±1.40 in f‐IMRT and from 13.38±0.98 in RA to $14.36\pm 1.28;\;D_{2\%}$ increased from 68.18±0.96 Gy in f‐IMRT and 68.09±0.84 Gy in RA to 68.61±0.87 Gy; and $V_{110\%}$ increased from 11.0%± 6.8% in f‐IMRT and 11.6%± 8.6% in RA to 14.3%± 6.8%. Although the $V_{95\%}$ and $D_{98\%}$ of f‐IMRT was better than that of RAx and RA (p<0.05), there was no statistical differences in the CI. There was no statistical difference in $V_{95\%}$ and $D_{98\%}$ for RAx and RA. Of all the ten cases, tumor coverage in RapidArc plans reached the equivalent clinical requirements in f‐IMRT, though the DVH data showed statistical differences in some parameters. In RapidArc, direct aperture optimization (DAO)^(16)^ was adopted in Eclipse, and due to mechanical limits of MLC motion, it is hard to realize manual adjustment of MLC in each aperture. Therefore, it is more difficult to control the high‐dose spots in RapidArc plans compared with f‐IMRT.

**Table 1:** PTV coverage analysis from the DVHs.

*Parameters*	*IMRT*	*RA*	*RAx*	*P*
CI	0.80±0.05	0.80±0.07	0.77±0.08	>0.05
HI	13.19±1.40	13.38±0.98	14.36±1.28	b,c
$D_{2\%}$ (Gy)	68.18±0.96	68.09±0.84.	68.61±0.87	b,c
$D_{98\%}$ (Gy)	60.27±0.48	60.06±0.47	60.00±0.52	a,b
$V_{95\%}(\%)$	98.6±0.7	98.3±0.9	98.1±0.9	a,b
$V_{110\%}(\%)$	11.0±6.8	11.6±8.6	14.3±6.8	b,c

Statistically significant differences (p<0.05) of paired t‐test analysis for three plans; a: IMRT vs. RA, b: IMRT vs. RAx, c: RA vs. RAx.

### B. Total lung dose

As summarized in Table 2, lung $V_{5}$ and $V_{10}$ in RAx plans were lower than that in f‐IMRT and RA plans (p<0.05). $V_{5}$ reduced from 50.9%± 9.8% in f‐IMRT and 51.4%± 10.8% in RA to 49.3%± 10.4%; and $V_{10}$ reduced from 39.6%± 7.5% in f‐IMRT and 38.5%± 7.1% in RA to 36.2%± 8.8%. Lung $V_{20}$ in RAx was lower than that of f‐IMRT and RA (p<0.05), while there was no statistical difference between f‐IMRT and RA. Lung $V_{30},V_{40},V_{50}$ and MLD in RAx were all higher than f‐IMRT and RA (p<0.05).

**Table 2:** Lung dose calculated from the DVHs.

*Parameters*	*IMRT*	*RA*	*RAx*	*P*
$V_{5}\;\text{Gy}\;(\%)$	50.9±9.8	51.4±10.8	49.3±10.4	b,c
$V_{10}\;\text{Gy}\;(\%)$	39.6±7.5	38.5±7.1	36.2±8.8	b
$V_{20}\;\text{Gy}\;(\%)$	21.6±4.1	20.7±3.2	21.8±4.1	c
$V_{30}\;\text{Gy}\;(\%)$	12.5±3.3	13.0±2.8	13.9±2.8	b,c
$V_{40}\;\text{Gy}\;(\%)$	7.9±2.7	8.1±2.7	8.9±2.6	b,c
$V_{50}\;\text{Gy}\;(\%)$	3.8±1.8	4.3±2.0	4.7±2.3	b,c
MLD (Gy)	12.03±1.85	12.02±1.76	12.16±1.86	b,c

Statistically significant differences (p<0.05) of paired t‐test analysis for three plans; a: IMRT vs. RA; b: IMRT vs. RAx; c: RA vs. RAx.

### C. B‐P

As summarized in Table 3, RA could not achieve the result of reducing low‐dose volume $(V_{3}$ and $V_{5}$) of healthy tissues, but RAx performed superior to the other two planning techniques in this aspect. $V_{3}$ of B‐P reduced form 86.3%±19.5% in f‐IMRT and 90.4%±19.7% in RA to $80.0\%\pm 15.5\%;V_{5}$ reduced from 77.9%±18.4% in f‐IMRT and 81.9%±19.5% in RA to $70.6\%\pm 16.2\%;V_{10}$ reduced from 62.0%±15.8% in f‐IMRT and 59.6%±16.0% in RA to 54.8%±14.1%.

**Table 3:** Dose to healthy tissues (B‐P) calculated from the DVHs.

*Parameters*	*IMRT*	*RA*	*RAx*	*P*
$V_{3}\;\text{Gy}\;(\%)$	86.3±19.5	90.4±19.7	80.0±15.5	a,b,c
$V_{5}\;\text{Gy}\;(\%)$	77.9±18.4	81.9±19.5	70.6±16.2	a,b,c
$V_{10}\;\text{Gy}\;(\%)$	62.0±15.8	59.6±16.0	54.8±14.1	b,c

Statistically significant differences (p<0.05) of paired t‐test analysis for three plans; a: IMRT vs. RA; b: IMRT vs. RAx; c: RA vs. RAx.

**Figure 1:**
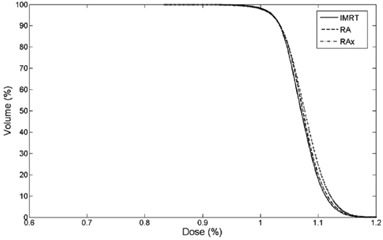
Average DVH of PTV for IMRT, RA and RAx.

**Figure 2:**
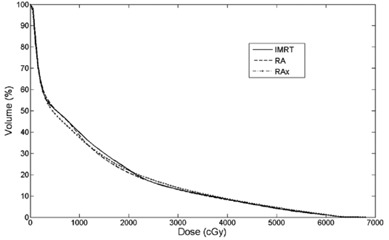
Average DVH of total lung for IMRT, RA and RAx.

**Figure 3:**
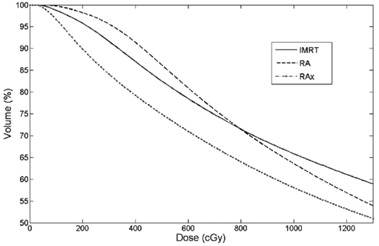
Average DVH of healthy tissues for IMRT, RA and RAx.

Average cumulative DVH for PTV, OARs and B‐P were built from the individual DVHs (Figs. 1–3). These histograms were obtained by averaging the corresponding volumes.

### D. Heart dose

In this study, most heart volumes were not involved in the radiation area. The results indicated that there were no differences in mean heart doses in three plans.

### E. Total MU and treatment time

As displayed in Table 4, compared with f‐IMRT (1174.8±144.6), the total MU was reduced by 31.6% to 803.8±122.2 in RA, and 37.3% to 736.2±186.9 in RAx. Because of X‐ray transmission through MLC, fewer MUs were needed, resulting in fewer low doses scattered in the radiation area. Therefore, IMAT technique is superior to f‐IMRT in controlling low‐dose volume, theoretically. The treatment time for each case in RA was 2.66 min, and was almost the same in RAx, while in f‐IMRT, it was 11.39±1.03 min, on average.

**Table 4:** Total MU and treatment time.

*Cases*	*Total MU*			*Time (min)*		
	*IMRT*	*RA*	*RAx*	*IMRT*	*RA* ^a^	*RAx*
1	1053	719	650	10.2	2.66	2.66
2	1081	956	764	10.5	2.66	2.66
3	1113	893	1171	11.1	2.66	3.3^b^
4	1202	691	792	12.0	2.66	2.66
5	1247	631	773	12.4	2.66	2.66
6	1057	934	831	10.1	2.66	2.66
7	1491	936	601	13.2	2.66	2.66
8	1288	697	520	12.5	2.66	2.66
9	1010	730	540	9.9	2.66	2.66
10	1206	851	720	12.0	2.66	2.66
Average	1174.8	803.8	736.2	11.39	2.66	2.66

a all fields rotated at the maximum speed

b fields rotated at a variable speed with the maximum dose rate.

**Figure 4:**
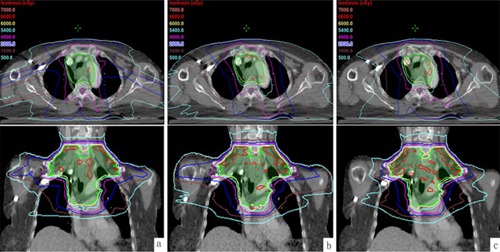
Isodose distributions on axial and coronal views of one case: a) IMRT; b) RA; c) RAx.

As Fig. 4 shows, three plans for one representative case were compared qualitatively in isodose distribution at axial and coronal views.

## IV. DISCUSSION

Since Brahme et al.^(17)^ put forward the IMRT technique, it has been widely applied in clinical treatment for many kinds of solid tumors. IMRT can achieve better tumor dose coverage, as well as sparing OAR and normal tissues. Generally, f‐IMRT plan adopted five to nine fixed fields, and it will cause relatively larger low‐dose volume on OAR and normal tissues which may lead to side effects, such as radiation pneumonitis,^(18)^ as well as inducing second primary cancer.^(19)^ The relative long treatment time of f‐IMRT will also increase patients’ discomfort. Although the effect of prolonging fraction time has not been clearly acknowledged in clinical radiotherapy, careful consideration should be given to the clinical treatment. Mu et al.^(20)^ concluded that the effects of prolonging fraction time were underestimated by biological models. The prolongation of the fraction time will spare tissues with a fast DNA healing, but there is also a risk for sparing tumors. It should be considered when IMRT is implemented in clinical treatment. Moiseenko et al.^(21)^ studied the IMRT time effect in three cell lines: Chinese hamster V79 fibroblasts, human cervical carcinoma SiHa and colon adenocarcinoma WiDr. In all cases, the cell survival results reached a consistent conclusion. Although Keall et al.^(22)^ and Paganetti^(23)^ obtained different results, they also suggested it ought to reduce the total treatment time in a fraction. Compared with f‐IMRT, RapidArc displayed a decrease of total treatment time by about 80%. The treatment was completed within 2.66–3.3 min without considering couch movement and other factors, which leads to less discomfort and better compliance from the patient. It could also reduce influences of body position movement, organ motion and volume change, and other uncertain factors on therapeutic effects. These advantages benefit hundreds of patients in our center who no longer need to wait for therapy, and daily QA takes less time. Furthermore, the relatively shorter treatment time could ensure the effect of rectification of setup error through on‐board imaging system. Shorter treatment time, higher dose rate (Varian 6MV‐X‐SRS mode, 1000 MU/min) and supports from image‐guide technique would make RapidArc more suitable for conducting hypofractionated accurate radiotherapy. Total MU of RapidArc was 30%–40% less than f‐IMRT. Besides, due to DAO algorithm during optimization, the total MLC motion scope was smaller than f‐IMRT, which could, to a certain extent, reduce MLC engines’ abrasions and maintenance cost of the accelerator. For a radiotherapy plan, while the target shape is complicated and dose constraints on OAR are strict, the number of aperture and MU increase correspondingly to meet the requirements, with less of an increase in RapidArc than in f‐IMRT. But if the total MU of RapidArc is close to f‐IMRT, there will be no advantage in dosimetry. The total MU in RapidArc plan of one case in this study was, respectively, 88.4% (RA) and 73.9% (RAx) of that in f‐IMRT, with most parameters of total lung and B‐P higher than f‐IMRT.

For RapidArc, because the gantry is always in rotation during the treatment, it cannot form a planar fluence. However, the optimization variables are the leaves position of MLC in each incidence direction, which is called aperture. Considering the degree of freedom in optimization selection, it is easy to obtain a better result for f‐IMRT by optimizing beamlets of each fixed field, rather than that for IMAT by optimizing angles and apertures in a rotation cycle. In Eclipse system, a fluence map is first obtained after optimization, and then converted into MLC apertures. Because the transmissions of MLC are only then taken into consideration, the low‐dose volume cannot be controlled under anticipation. DAO was adopted as optimization algorithm in RapidArc, so the final results come closer to optimization anticipation. Wagner et al.^(24)^ found that f‐IMRT was better than RapidArc in both PTV coverage and OAR sparing for glioma. If PTV was large enough, RapidArc could be inferior to f‐IMRT on PTV coverage and lead to a larger low‐dose volume. The results in our study supported the conclusion. Alternatively, the RAx plans had B‐P volume reduced. According to the studies from Liu et al.^(18)^ and Nutting et al.,^(25)^ f‐IMRT could reduce lung $V_{20},V_{30}$ and MLD in lung and esophageal cancer, but an increase of lung $V_{5}$ and $V_{10}$ were also observed. Recent Studies^(^ ^18^ ^,^ ^26^ ^–^ ^29^ ^)^ indicated that there was a certain relationship between the incidence/degree of radiation pneumonia and low‐dose irradiation (< 5 Gy). Gopal et al.^(28)^ pointed out that a small dose of radiation to a large volume of lung could be much worse than a large dose to a small volume, in functional lung damage. Because of the high utilization rate of X‐ray and less apertures and MU, IMAT can theoretically reduce the low‐dose volume in radiation area. This was supported by the data of healthy tissues (B‐P) in this study. With less total MUs, it could produce less X‐ray transmission of MLC. The RAx was designed primarily to reduce the volume of organs involved in radiation area and achieve similar treatment effect with RA and f‐IMRT. Although the lung volume > 20 Gy and MLD increased slightly, it was still within the clinical tolerance.^(^ ^27^ ^,^ ^30^ ^)^ On the other hand, the advantage of RAx was reducing low‐dose volume (especially for B‐P). The value of $V_{5}$ reduced on average 1.6% from f‐IMRT and 2.1% from RA, as shown in Table 2 (p<0.05). Due to the absence of an optimal threshold of $V_{5}$, it's considered to be better with a lower value, with the value of $V_{20}$ and MLD in a safer clinical tolerance. And the clinical significance of this difference need to be further investigated. In this study, the lung involved was the upper part in all cases. Although Yorke et al.^(29)^ had concluded in their study that there was no correlation between dose and pneumonitis, the value of $V_{5}$ here was not to verify the conclusion, but a parameter, as the same as that in B‐P, to prove the reduction of low‐dose volume involved. As RAx was based on RA, it could reduce the low‐dose volume, with the equivalent PTV coverage. Furthermore, RapidArc was not inferior to f‐IMRT on dosimetric metrics, summarized in Table 2. For these cases, the radiation targets were located in mediastinum centrally and symmetrically. Therefore, there's no significant difference between right and left lung doses.

These results differ from conventional IMRT in Eclipse, which optimizes intensity map, while the DAO algorithm optimizes the shapes and weights of apertures. The additional number of optimization variables including mechanical constraints make the DAO process complicated and relatively time‐consuming. After Otto^(31)^ and Luan et al.^(32)^ had succeeded in conducting improvement on DAO algorithm to achieve higher efficiency, and with upgrade of hardware, the optimizing time had been greatly reduced. In this study, the total optimization time of RapidArc plans were three to five times as that of f‐IMRT. Furthermore, generating a good RapidArc plan depended to a great extent on experience. In a RapidArc plan, the number of fields, starting and ending rotation angles, collimator angle, couch angle, jaw positions, optimization constraints, and control of optimization process have to be further studied and summarized by physicists. Vanetti E et al.^(33)^ found that RapidArc plan could not meet the dosimetric or clinical requirement for head and neck tumor using only one arc. However, the brainstem and parotids could be well protected in the RapidArc plans using two arcs. Our initial study demonstrated that one arc (one field rotated fully) was not sufficient to achieve the dosimetric goals compared to two arcs for cervical esophageal cancer. The key differences are shown as Table 5. Therefore, two RapidArc arcs (RA) and an improved method based on it (RAx) were adopted in this study. However, Wang et al.^(34)^ had recently developed a new algorithm (AMRT) using one arc and achieved ideal dose distribution. It is assumed that, using the algorithm, the treatment time would be reduced, more or less, with an ideal tumor coverage and dose distribution.

**Table 5:** Comparison of RA‐1 and RA‐2 from DVH in the previous study.

*Parameters*	*RA‐1* ^a^	*RA‐2*
PTV		
CI	0.80±0.05	0.80±0.07
HI(%)	16.2±1.4	13.4±1.0
$V_{110\%}(\%)$	20.2±8.3	11.6±8.6
Total‐lung		
$V_{5}\;\text{Gy}\;(\%)$	51.5±10.7	51.4±10.8
$V_{10}$ Gy [%]	41.8±9.3	38.5±7.1
$V_{20}\;\text{Gy}\;(\%)$	24.7±4.8	20.7±3.2
$V_{30}\;\text{Gy}\;(\%)$	14.5±3.5	13.0±2.8
MLD (Gy)	12.83±2.03	12.02±1.76
B‐P		
$V_{5}\;\text{Gy}\;(\%)$	83.2±19.7	81.9±19.5
$V_{10}\;\text{Gy}\;(\%)$	65.0±17.1	59.6±16.0
Total MU	709.7±101.8	803.8±122.2
Time	1.9±0.3 ^b^	2.66

a RA‐1 were set one arc, RA‐2 were set two arcs

b RA‐1 rotated at a variable speed with the maximum dose rate.

## V. CONCLUSIONS

RapidArc could be delivered to achieve the similar tumor coverage as f‐IMRT and effectively sparing OARs. The improved method (RAx) could reduce low‐dose volume of the tissues to a certain extent. It could be served as an alternative therapeutic method for cervical esophageal cancer. To summarize, the RapidArc technology exhibited a remarkable ability in delivering with much shorter time and less MU.

## ACKNOWLEDGMENTS

Thanks to Kun Yang and Jun Ma for their contributions to the writing of this paper.
